# Supplementary material for: Polyprotein processing and intermolecular interactions within the viral replication complex spatially and temporally control norovirus protease activity
Source: J Biol Chem. 2019 Jan 15;294(11):4259–71. doi: 10.1074/jbc.RA118.006780 (PMC6422069; doi:10.1074/jbc.RA118.006780)
Supplement: Supporting Information [file supp_294_11_4259__index.html]

Polyprotein processing and intermolecular interactions within the viral replication complex spatially and temporally control norovirus protease activity — Regulation of norovirus protease activity during infection — Polyprotein processing and intermolecular interactions within the viral replication complex spatially and temporally control norovirus protease activity — Regulation of norovirus protease activity during infection — Supporting Information 

# Polyprotein processing and intermolecular interactions within the viral replication complex spatially and temporally control norovirus protease activity

## Supporting Information

- Supplementary figures - Supplementary figures
- Table S1 - Supplementary table illustrating the expected molecular masses of the various precursor proteins.
