# Supplementary material for: Polyprotein processing and intermolecular interactions within the viral replication complex spatially and temporally control norovirus protease activity
Source: J Biol Chem. 2019 Jan 15;294(11):4259–71. doi: 10.1074/jbc.RA118.006780 (PMC6422069; doi:10.1074/jbc.RA118.006780)
Supplement: Supporting Information [file supp_RA118.006780_142048_0_supp_268506_pldtjh.pdf]

Figure S1. *Confirmation of precursor identity in MNV-infected cell lysates.* Lysates from BV-2 cells mock- or infected with MNV at MOI 10 and harvested at 9h post-infection, were compared to HEK-293T cell lysates mock- or transfected with plasmids expressing stable forms of the various precursors. Precursors were visualized by anti-NS6 and anti-NS5 antisera to assess co-migration. Note that the addition of a FLAG-tag on the precursors adds at additional 1.14kDa which results in some discrepancy in the migration of the smaller precursors. All experiments were repeated two times.

Fig S1.

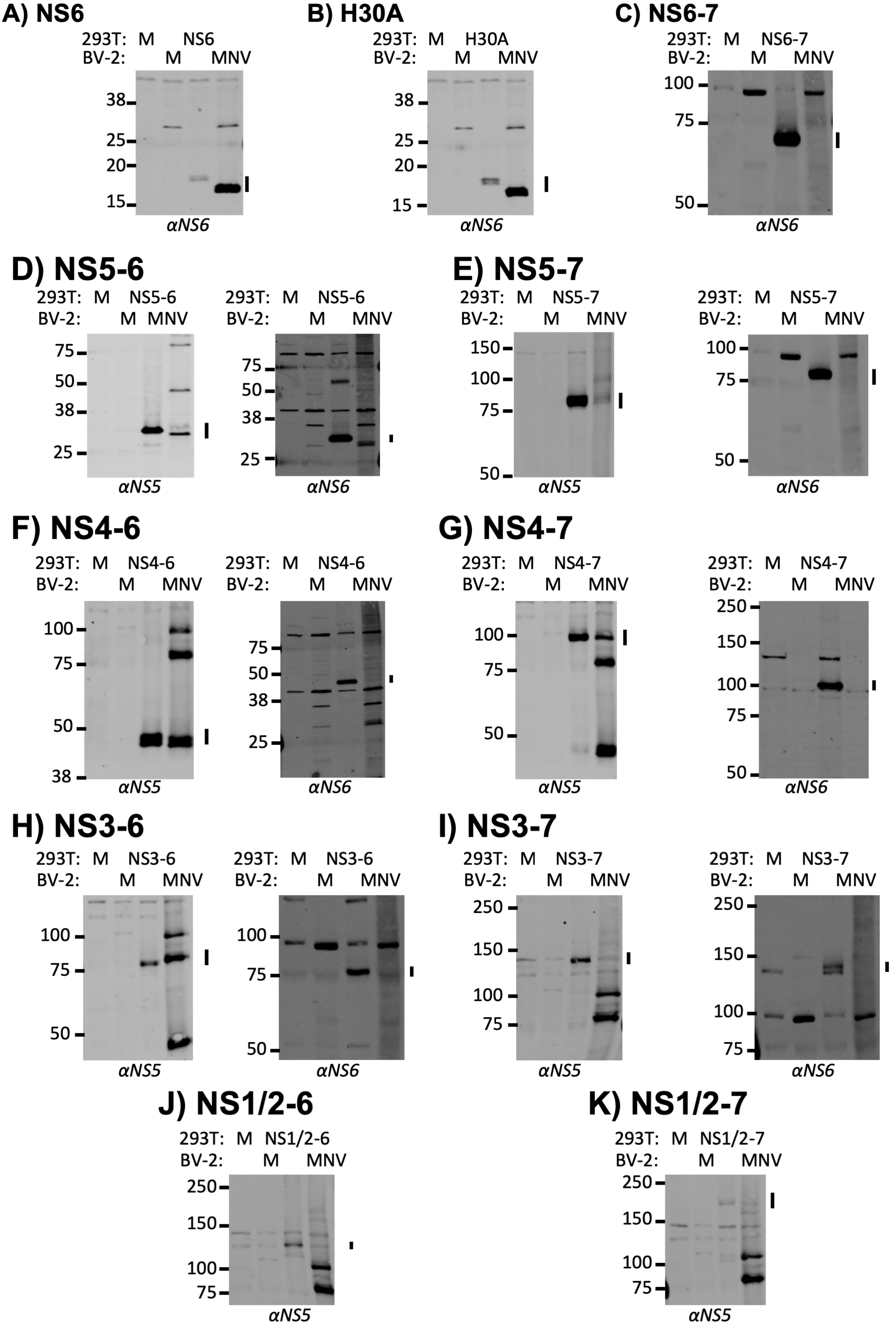

A) NS6
293T: M NS6
BV-2: M MNV
38
25
20
15
αNS6
B) H30A
293T: M H30A
BV-2: M MNV
38
25
20
15
αNS6
C) NS6-7
293T: M NS6-7
BV-2: M MNV
100
75
50
αNS6
D) NS5-6
293T: M NS5-6
BV-2: M MNV
75
50
38
25
αNS5
293T: M NS5-6
BV-2: M MNV
75
50
38
25
αNS6
E) NS5-7
293T: M NS5-7
BV-2: M MNV
150
100
75
50
αNS5
293T: M NS5-7
BV-2: M MNV
100
75
50
αNS6
F) NS4-6
293T: M NS4-6
BV-2: M MNV
100
75
50
38
αNS5
293T: M NS4-6
BV-2: M MNV
75
50
38
25
αNS6
G) NS4-7
293T: M NS4-7
BV-2: M MNV
100
75
50
αNS5
293T: M NS4-7
BV-2: M MNV
250
150
100
75
50
αNS6
H) NS3-6
293T: M NS3-6
BV-2: M MNV
100
75
50
αNS5
293T: M NS3-6
BV-2: M MNV
100
75
50
αNS6
I) NS3-7
293T: M NS3-7
BV-2: M MNV
250
150
100
75
αNS5
293T: M NS3-7
BV-2: M MNV
250
150
100
75
αNS6
J) NS1/2-6
293T: M NS1/2-6
BV-2: M MNV
250
150
100
75
αNS5
K) NS1/2-7
293T: M NS1/2-7
BV-2: M MNV
250
150
100
75
αNS5

Figure S2. *Cleavage of the FRET sensor by norovirus protease precursors in vitro.* Incubation of unlabeled *in vitro* translated norovirus protease precursors with ^{35}S Methionine-labeled *in vitro* translated substrate demonstrates cleavage of the FRET sensor. All experiments were repeated two times.

Fig S2.

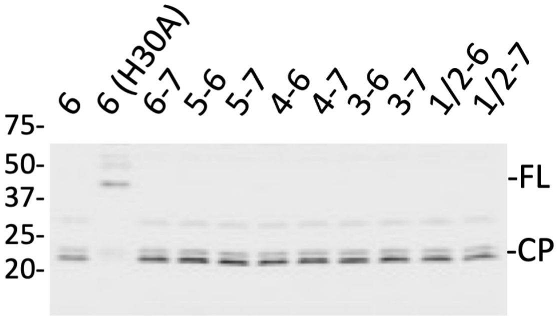

Figure S3. *Confocal microscopy of N-terminal norovirus protease precursors.* HeLa-CD300lf cells were transfected with plasmids expressing NS3-6, NS3-7, NS1/2-6 and NS1/2-7. At 18h post-transfection the cells were harvested and precursors visualized by anti-NS6 staining. Nuclei were visualized with DAPI. All experiments were repeated two times, and 3 fields of view imaged for each protease precursor. One representative image for each condition is shown.

Fig S3.

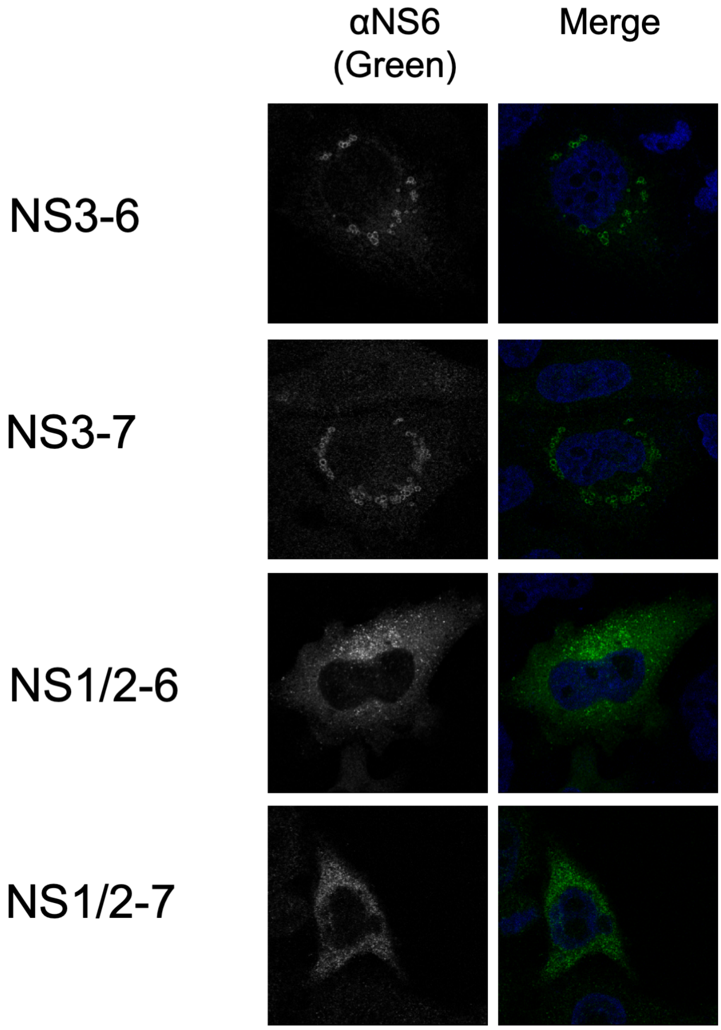

Figure S4. *Western blotting of MNV LUMIER assay reagents confirms expression.* Western blotting of lysates prepared from HEK-293T cells transfected with A) Renilla-luciferase or B) Protein-A fusions of the various MNV proteins confirms their expression and migration at the expected size. Samples were harvested at 24h post-transfection. Lumier experiments were performed in quadruplicate.

Fig S4.

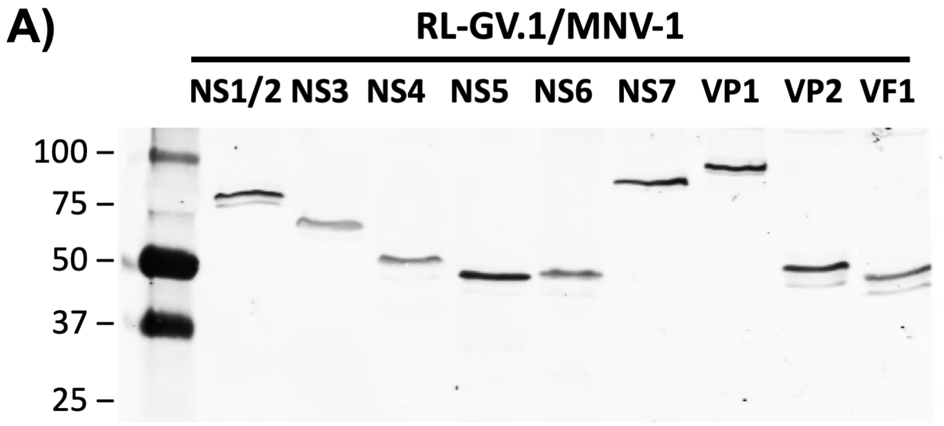
A)
RL-GV.1/MNV-1
NS1/2 NS3 NS4 NS5 NS6 NS7 VP1 VP2 VF1
100
75
50
37
25

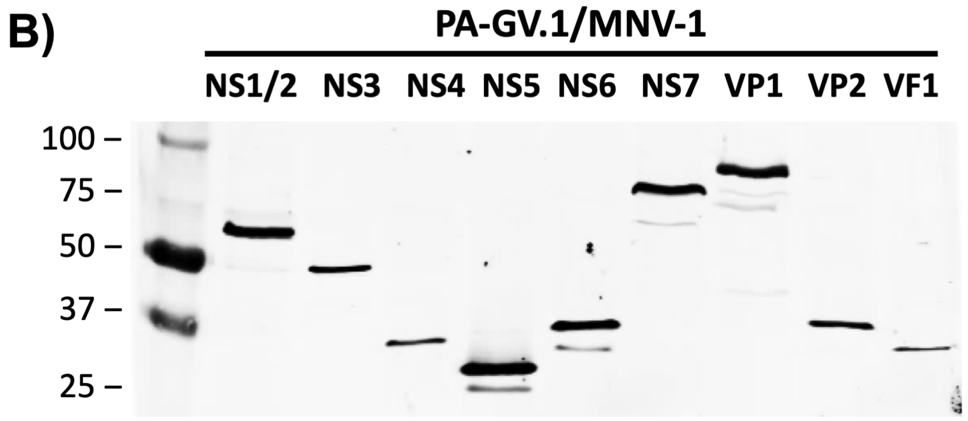
B)
PA-GV.1/MNV-1
NS1/2 NS3 NS4 NS5 NS6 NS7 VP1 VP2 VF1
100
75
50
37
25

Figure S5. *LUMIER assay reveals GI.1 replication complex interactions are conserved between GI.1 and MNV.* A) HEK-293T cells expressing Protein-A and Renilla luciferase fusions of the various GI.1 human norovirus proteins were used for LUMIER analysis to identify protein:protein interactions. The numbers are robust z-scores. Positive protein:protein interactions are coloured by the strength of interaction with weak interactions showing in pale yellow, with the strongest interactions in purple. Western blotting of lysates prepared from HEK-293T cells transfected with B) Renilla-luciferase or C) Protein-A fusions of the various GI.1 norovirus proteins confirms their expression and migration at the expected size. Samples were harvested at 24h post-transfection. Lumier experiments were performed in quadruplicate.

Fig S5.

A)

NV GI.1 — Protein A fusion proteins (columns); Renilla luciferase fusion proteins (rows)

NV GI.1	NS1/2	NS3	NS4	NS5	NS6	NS7	VP1	VP2
NS1/2	29.5	-0.9	21.5	0.6	1.7	4.1	0.3	-2.0
NS3	1.5	1.6	1.9	-0.4	0.3	0.1	-0.7	-0.7
NS4	37.7	-0.4	5.5	0.1	-3.2	-0.8	2.1	-2.5
NS5	-1.6	0.2	-0.6	1.3	1.6	11.5	0.6	-1.0
NS6	1.3	-2.4	-1.7	-2.1	-2.0	-2.3	0.0	-2.3
NS7	4.7	-1.3	-1.2	4.3	0.4	6.5	6.5	2.0
VP1	3.9	-0.1	2.0	1.5	-0.1	4.7	32.4	4.6
VP2	1.6	-0.7	-0.5	1.7	2.8	1.8	17.9	13.8

RZ-score scale					
<3	3-6	6-9	9-12	12-15	>15

B)

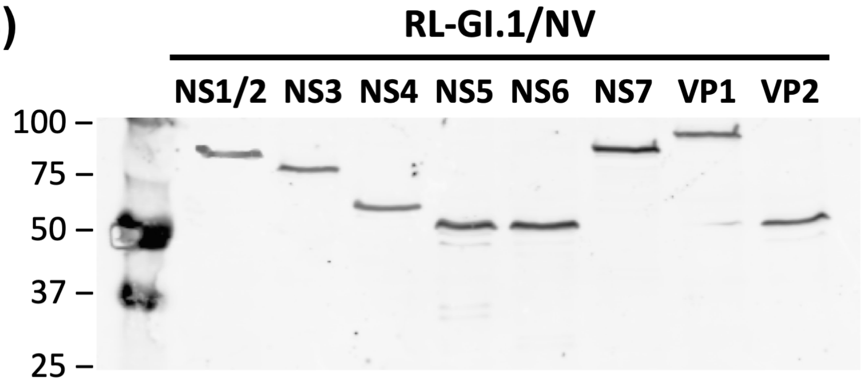

C)

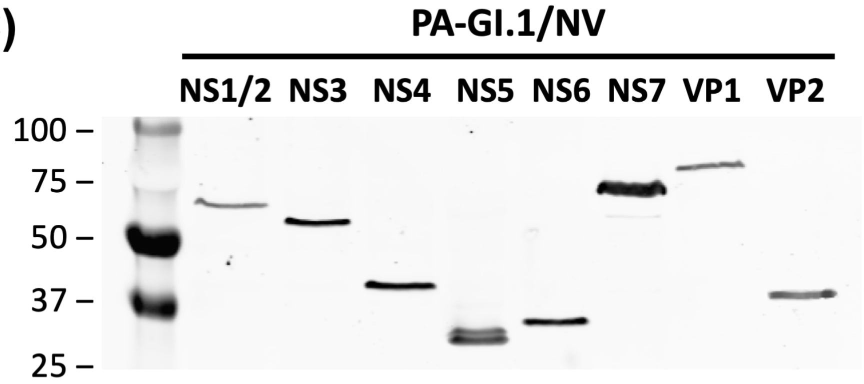

Figure S6. *LUMIER assay reveals GII.4 replication complex interactions are conserved between GII.4 and MNV.* A) HEK-293T cells expressing Protein-A and Renilla luciferase fusions of the various GII.4 human norovirus proteins were used for LUMIER analysis to identify protein:protein interactions. The numbers are robust z-scores. Positive protein:protein interactions are coloured by the strength of interaction with weak interactions showing in pale yellow, with the strongest interactions in purple. Western blotting of lysates prepared from HEK-293T cells transfected with B) Renilla-luciferase or C) Protein-A fusions of the various GII.4 norovirus proteins confirms their expression and migration at the expected size. Samples were harvested at 24h post-transfection. Lumier experiments were performed in quadruplicate.

Fig S6.

A)

MD GII.4	Protein A fusion proteins							
Renilla luciferase fusion proteins	NS1/2	NS3	NS4	NS5	NS6	NS7	VP1	VP2
NS1/2	27.3	1.6	27.8	0.8	1.2	3.2	1.4	1.5
NS3	1.1	1.0	-0.3	0.7	-0.1	0.3	0.0	0.0
NS4	24.9	-0.6	3.5	1.6	0.6	0.4	1.5	1.1
NS5	2.3	-0.3	-2.7	-0.6	-0.7	7.7	0.7	1.5
NS6	1.5	-1.2	-0.6	-0.6	0.8	-0.5	-0.9	-0.2
NS7	8.9	1.6	2.2	3.2	0.8	7.1	13.3	-1.8
VP1	5.9	-0.1	-0.7	2.3	0.6	5.4	8.6	13.9
VP2	2.0	1.3	-1.4	1.2	1.0	0.9	10.0	10.3

RZ-score scale	<3	3-6	6-9	9-12	12-15	>15

B)

RL-GII.4/MD145

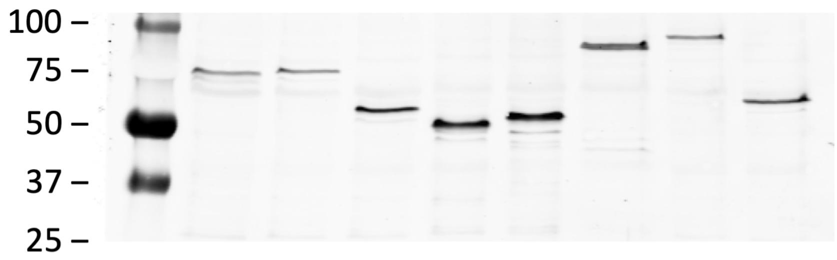

C)

PA-GII.4/MD145

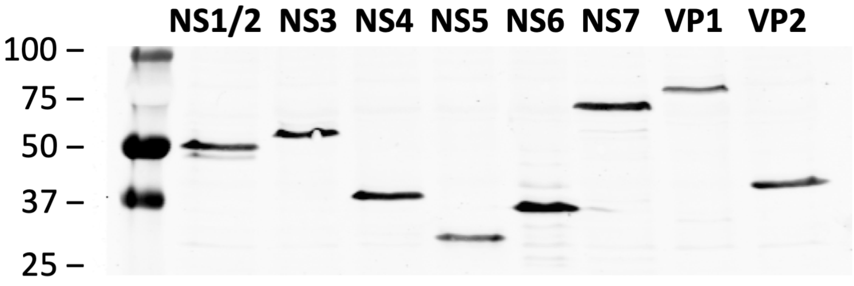

Figure S7. *Replicate data relating to Figure 7.* Polyprotein-FRET fusions mutants were transfected into BSR-T7 cells to function as trans-cleavage substrates. WT or proteolytically-inactive full length clone (MNV-FLC, MNV-H30A) was provided *in trans* to determine cleavage efficiency. Samples were harvested at 18h post-transfection. A) N-terminal, B) C-terminal and C) Internal deletions are shown. Substrate cleavage was assessed using anti-GFP antisera. ORF1-FRET-WT was used as a positive control (+). For clarity the position of either the full length (Mock cells) or fully-processed cleavage product (MNV infected cells) is highlighted with a red asterisk (*).

Fig S7.

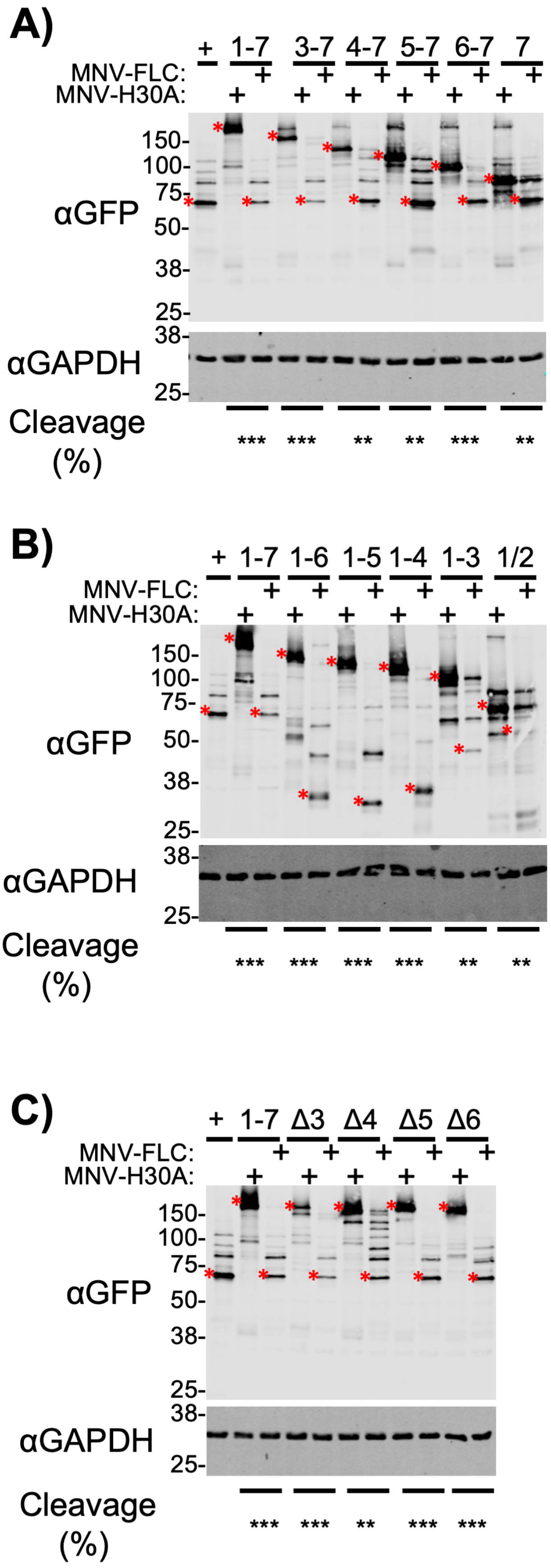
A)
+
1-7
3-7
4-7
5-7
6-7
7
MNV-FLC:
MNV-H30A:
150-
100-
75-
50-
38-
25-
αGFP
38-
αGAPDH
25-
Cleavage (%)
*** *** ** ** *** **
B)
+
1-7
1-6
1-5
1-4
1-3
1/2
MNV-FLC:
MNV-H30A:
αGFP
αGAPDH
Cleavage (%)
*** *** *** *** ** **
C)
+
1-7
Δ3
Δ4
Δ5
Δ6
MNV-FLC:
MNV-H30A:
αGFP
αGAPDH
Cleavage (%)
*** *** ** *** ***

Table S1. *Predicted molecular weights of norovirus proteins and precursor forms.* The first tab lists all the potential norovirus proteins and precursors that can be derived from the polyprotein. The second and third tabs list all the NS5- or NS6-containing precursors ranked by molecular weight from largest to smallest. Predicted molecular weight was calculated using the ExPASy server ProtParam tool.
